# Soil bacterial communities in three rice-based cropping systems differing in productivity

**DOI:** 10.1038/s41598-020-66924-8

**Published:** 2020-06-17

**Authors:** Min Huang, Alin Tian, Jiana Chen, Fangbo Cao, Yumei Chen, Longsheng Liu

**Affiliations:** 1 Crop and Environment Research Center, College of Agronomy, Hunan Agricultural University, Changsha, 410128 China; 2 Department of Crop Cultivation, Hengyang Academy of Agricultural Sciences, Hengyang, 421101 China

**Keywords:** Microbial ecology, Agroecology

## Abstract

Soil microorganisms play an important role in determining productivity of agro-ecosystems. This study was conducted to compare diversity, richness, and structure (relative abundance at the phylum level) of soil bacterial communities among three rice-based cropping systems, namely, a winter fallow-rice-rice (FRR), green manure (Chinese milk vetch)-rice-rice (MRR), and oilseed rape-rice-rice (ORR), in which MRR and ORR had significantly higher productivity than FRR. A 16S rRNA gene sequence analysis showed that no significant differences were observed in diversity and richness indices (observed species, Shannon, Simpson, Chao1, abundance-based coverage estimators, and phylogeny-based metrics) of soil bacterial communities among the three cropping systems. However, relative abundances of dominant phyla in soil bacterial communities, including Proteobacteria, Acidobacteria, Nitrospirae, Gemmatimonadetes, and Verrucomicrobia, were significantly different among the three cropping systems. In particular, a significant reduction in the relative abundance of Nitrospirae was observed in both MRR and ORR compared with FRR. These results indicate that bacterial community structure was affected by cropping systems in the tested paddy soils. Based on the results of our studies and existing knowledge bases, we speculate that benefits to rice yield may be obtained by reducing the relative abundance of Nitrospirae and increasing the ratio of abundances of Proteobacteria/Acidobacteria in paddy soils.

## Introduction

Rice is the major staple food crop for many populations in the world, especially in Asia where production and consumption accounts for more than 90% of the rice worldwide^1^. The intensification of rice cropping systems has contributed greatly to ensuring food security in Asia^2^, and a further increase in cropping intensity is an important approach to achieving greater food security in the future^3^. However, the long-term continuous rice cropping systems have posed a challenge to sustainable land productivity^4,5^.

Well-planned cropping systems are expected to promote effective use of natural resources and maintain land productivity in the long-term to achieve greater crop yields and sustainability of cropping systems^6^. In recent years, the role of soil microorganisms in improving productivity of agro-ecosystems has gained more attention^7^, and more recent evidence indicates that cropping systems influence rice yield by altering soil bacterial communities^8^.

Double-season rice cropping system is a major intensive cropping system in China. In the double-season rice cropping regions, farmers are strongly recommended to grow green manure or oilseed rape in the winter season to improve rice yields. Our recent study also supports this recommendation^9^. Here, we hypothesized that growing green manure and oilseed rape in the winter season would alter soil bacterial communities in double-season rice paddies. To test this hypothesis, we collected soil samples from the experimental field of Huang *et al*.^9^. to determine cropping system effects on diversity, richness, and structure (relative abundance at phylum level) of the soil bacterial communities.

## Results and Discussion

There were no significant differences within each diversity and richness index among soil bacterial communities of the FRR, MRR, and ORR cropping systems (*p* > 0.05; Table 1). Thus, diversity and richness of the soil bacterial communities were not significantly affected by cropping systems in this study. This finding, though unexpected, may be explained by a previous study by Xuan *et al*.^8^. They observed that the effects of cropping systems (rice-rice-rice, rice-maize-rice, rice-mungbean-rice, and rice-mungbean-maize) on bacterial community diversity and richness in a paddy soil differed between sampling times; cropping systems significantly affected Shannon and Chao1 indices of soil bacterial communities from soils sampled mid-season of a crop rotation but not those of soils sampled after rice harvest. In our study, the tested soils unaffected by cropping system were sampled after rice harvest. This suggests that further investigations are required to determine the effects of cropping systems on dynamic changes in diversity and richness of bacterial communities in paddy soils.

**Table 1:** Bacterial community diversity and richness in subtropical paddy soils sampled from three cropping systems.

Index^a^	Cropping system^b^			*p*-value
	FRR	MRR	ORR	
Observed species	2710	2741	2709	0.872
Shannon	9.31	9.31	9.23	0.441
Simpson	0.993	0.994	0.991	0.116
Chao1	3024	3154	3046	0.681
ACE	3091	3232	3120	0.606
PD whole tree	217	219	217	0.945

^a^ACE, abundance-based coverage estimators; PD whole tree, phylogeny-based metrics.

^b^FRR, fallow-rice-rice; MRR, green manure-rice-rice; ORR, oilseed rape-rice-rice.

**Figure 1:**
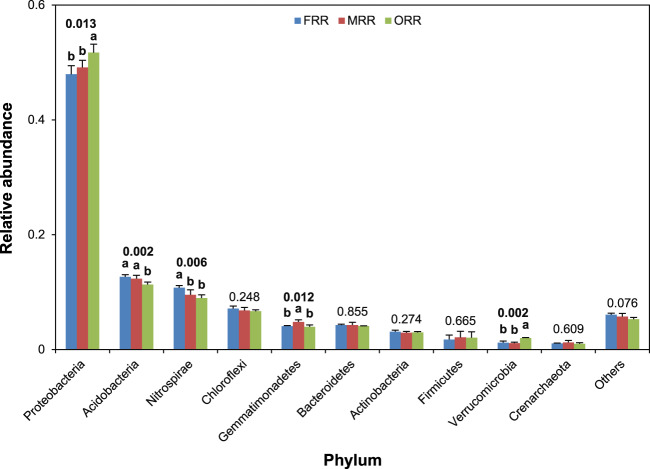
Relative abundances of bacterial phyla in paddy soils from three cropping systems FRR (fallow-rice-rice), MRR (green manure-rice-rice), and ORR (oilseed rape-rice-rice). Data are means and SE (*n* = 4). Values above bars are *p*-values. Different letters denote significant differences at *p* < 0.05 by multiple comparisons among cropping systems.

The top 10 phyla of bacterial communities according to their relative abundances in the soil across three cropping systems were Proteobacteria, Acidobacteria, Nitrospirae, Chloroflexi, Gemmatimonadetes, Bacteroidetes, Actinobacteria, Firmicutes, Verrucomicrobia, and Crenarchaeota (Fig. 1). Previous studies also reported eight of our top 10 phyla (the exceptions were Gemmatimonadetes and Crenarchaeota) in their top 10 phyla of bacterial communities in paddy soils of rice-rice-rice, rice-maize-rice, rice-mungbean-rice, rice-mungbean-maize, and FRR cropping systems^8,10^. This indicates that the dominant phyla of bacterial communities are relatively stable in paddy soils across cropping systems.

Rankings and relative abundances of the dominant phyla of soil bacterial communities differed between findings of the present (Fig. 1) and previous studies^8,10^. For example, Nitropirae respectively ranked 8th and 3rd among soil bacterial communities in this and Yin *et al*.’s study^10^, and the respective average relative abundances of Proteobacteria were 0.50 and 0.33 in this and Xuan *et al*.’s study^8^. Differences in environmental conditions (soil and climate) and crop management practices may explain the inconsistencies in rank and abundances of phyla^11,12^, but the specific reasons are unclear.

Relative abundances of Proteobacteria, Acidobacteria, Nitrospirae, Gemmatimonadetes, and Verrucomicrobia in soil bacterial communities were significantly different among the FRR, MRR, and ORR cropping systems (*p* < 0.05; Fig. 1). Compared with FRR, MRR had 11% lower relative abundance of Nitrospirae and 18% higher relative abundance of Gemmatimonadetes; ORR had 8–65% higher relative abundances of Proteobacteria and Verrucomicrobia and 11–17% lower relative abundances of Acidobacteria and Nitrospirae. These results indicate that the structure of soil bacterial communities was significantly affected by cropping systems in this study. Based on both the abundance and diversity and richness data, we suspect that interspecific competition is likely responsible for the insignificant effects of cropping systems on the diversity and richness of soil bacterial communities.

In conclusion, this study determined altered soil bacterial community structure among three rice-based cropping systems differing in productivity. Briefly, compared with FRR, the higher rice yield in MRR was accompanied by a decrease in relative abundance of Nitrospirae and an increase in relative abundance of Gemmatimonadetes, while the higher rice yield in ORR was accompanied by increases in relative abundances of Proteobacteria and Verrucomicrobia and decreases in relative abundances of Acidobacteria and Nitrospirae. These findings suggest that benefits to rice yield may be obtained by strategically shaping the soil bacterial community structure.

**Figure 2:**
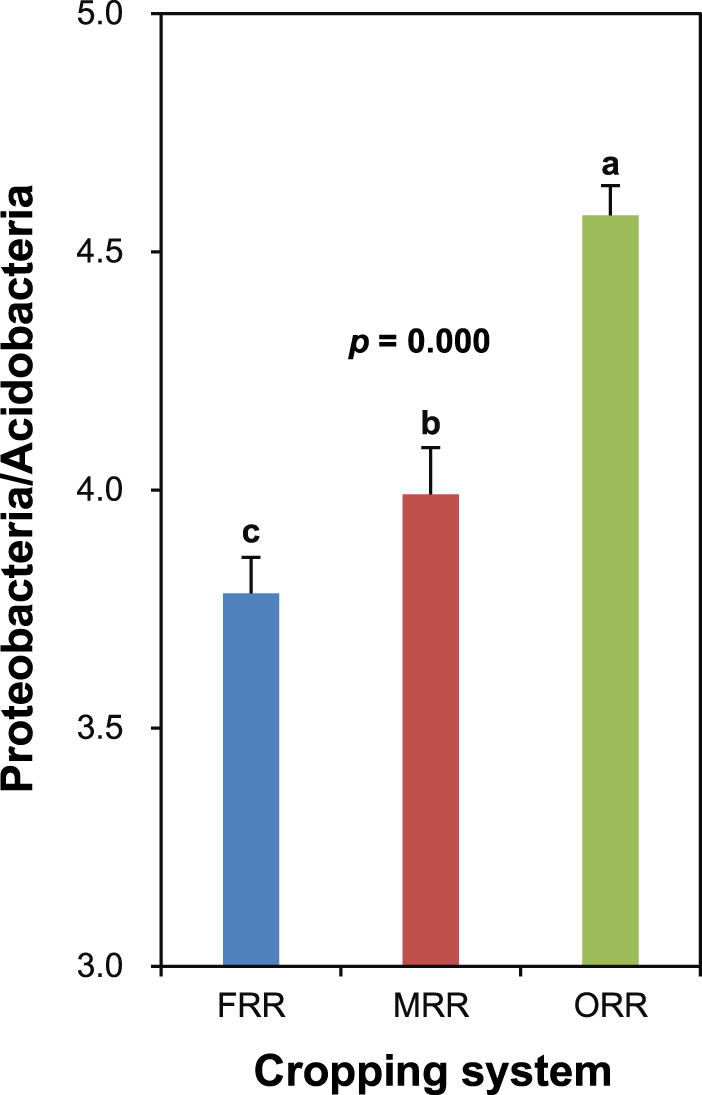
Ratios of abundances of Proteobacteria/Acidobacteria in paddy soils from three cropping systems FRR (fallow-rice-rice), MRR (green manure-rice-rice), and ORR (oilseed rape-rice-rice). Ratios were calculated by dividing the relative abundance of Proteobacteria by the relative abundance of Acidobacteria. Data are means and SE (*n* = 4). Different letters denote significant differences at *p* < 0.05 by multiple comparisons among cropping systems.

Based on the results of our present and previous studies and those of others, we speculate that reducing the relative abundance of Nitrospirae and increasing the ratio of abundances of Proteobacteria/Acidobacteria in paddy soils may be beneficial to improve rice yield. On one hand, a lower relative abundance of Nitrospirae was observed in both MRR and ORR with higher rice yield than in FRR with lower rice yield in our studies. Nitrospirae is a phylum of nitrification bacteria in soils, which plays critical role in both the ammonia-oxidizing and nitrite-oxidizing processes^13^. A reduction in Nitrospirae abundance in the soil may reduce nitrification, and consequently increase availability of ammonia nitrogen (the main nitrogen source for rice) and decrease nitrogen loss through leaching and denitrification^14^, and finally improve nitrogen uptake and grain yield in rice^15^. On the other hand, abundances of Proteobacteria and Acidobacteria are related to the nutrient status of soils, and high ratios of abundances of Proteobacteria/Acidobacteria are indicative of copiotrophic soils^16^. In the present study, the significantly higher abundance of Proteobacteria or significantly lower abundance of Acidobacteria resulted in a significantly higher ratio of abundances of Proteobacteria/Acidobacteria in both MRR and ORR than in FRR (Figs. 1 and 2), suggesting that the soil nutrient status was improved in both MRR and ORR with higher rice yield compared to FRR with lower rice yield. In this regard, it has been documented that growing green manure of Chinese milkvetch can improve nitrogen recovery and conservation in the rice paddy soil^17^, while growing oilseed rape can increase soil organic matter and nitrogen (especially the available nitrogen) contents^18^. In addition, chemical fertilizers (120 kg N ha^−1^, 67.5 kg P_2_O_5_ ha^−1^, and 120 kg K_2_O ha^−1^) were applied to the oilseed rape crop in ORR^9^, which might also affect the nutrient status of soils and the growth and productivity of subsequent rice crops. The results of our studies highlight that further investigations are needed to gain a greater fundamental understanding of relationships among the structure of soil bacterial communities, soil nutrient status, and crop physiological processes in rice paddies. Such investigations will provide useful information to identify the specific soil microorganisms responsible for the high productivity of rice paddies.

## Methods

### Soil sampling

The experimental field of Huang *et al*.^9^ is located at Hengyang (26°53′ N, 112°28′ E), Hunan Province, China. Three rice-based cropping systems were established in the experimental field from 2014 to 2019 where two seasons of rice crops were preceded by a winter season of either a (1) fallow field (winter fallow-rice-rice, FRR), (2) crop of green manure of Chinese milkvetch (green manure-rice-rice, MRR), or (3) crop of oilseed rape (oilseed rape-rice-rice, ORR) in each cropping cycle. The cropping systems were laid out in a randomized complete-block design with four replications and a plot size of 45 m^2^. Averaged rice yield across cropping cycles was significantly higher in MRR and ORR than in FRR. The detailed information of the experiment and yield data can be found in Huang *et al*.^9^

At seven days after the second-season rice harvest in 2019, fifteen points were randomly selected in each plot to collect soil samples from the upper 20-cm layer with a flame-sterilized soil corer (inner diameter = 2 cm). The soil samples from each plot were composited after removing visible plant residues and gravel. All composited soil samples were placed in sterilized sealed bags, brought to the laboratory in an ice box, and stored in an ultra-cold freezer at –70 °C until assayed.

### Bacterial community assay

A 16S rRNA gene sequence analysis was conducted to assay bacterial communities of each soil sample. DNA extraction and 16 S rRNA gene sequencing were performed according to the procedures described by Yin *et al*.^10^. Quality-filtered and non-chimeric sequences were clustered to generate operational taxonomic units (OTUs) at a similarity level of 97% using UPARSE (v7.0.1090)^19^. Taxonomic information of OTUs was annotated against the GreenGene Database^20^ using the RDP classifier algorithm (v2.2)^21^.

Bacterial community diversity and richness indices, including observed species, Shannon, Simpson, Chao1, abundance-based coverage estimators (ACE), and phylogeny-based metrics (PD whole tree), were calculated using QIIME (v1.9.1)^22^. Relative abundance of each phylum was calculated by dividing the number of OTUs affiliated with a phylum by the total number of OTUs.

### Statistical analysis

Data were analysed by analysis of variance (ANOVA) using Statistix 8.0 analytical software (Tallahassee, FL, USA). The statistical significance level of ANOVA was set at *p* < 0.05. The variables with significant differences were subjected to multiple comparisons using the least significant difference (LSD) test at *p* < 0.05.

## Data Availability

All data generated or analysed during this study are included in the article.

## References

[1] Muthayya S, Sugimoto JD, Montgomery S, Maberly GF (2014). An overview of global rice production, supply, trade, and consumption. Ann. N. Y. Acad. Sci..

[2] Buresh, R. J., Larazo, W. M., Laureles, E. V., Samson, M. I. & Pampolino, M. F. Sustainable soil management in lowland rice ecosystems in *Organic-based agriculture for sustained soil health and productivity* (eds. Javier, E. F., Mendoza, D. M. & dela Cruz, N. E) 116–125 (Central Luzon State University, 2005).

[3] Ray DK, Foley JA (2013). Increasing global crop harvest frequency: recent trends and future directions. Environ. Res. Lett..

[4] Joshi AK, Chand R, Arun B, Singh RP, Ortiz R (2007). Breeding crops for reduced-tillage management in the intensive, rice-wheat systems of South Asia. Euphytica.

[5] Ladha JK (2003). How extensive are yield declines in long-term rice-wheat experiments in Asia?. Field Crops Res..

[6] Zegada-Lizarazu W, Monti A (2011). Energy crops in rotation. A review. Biom. Bioenergy.

[7] Welbaum GE, Sturz AV, Dong Z, Nowak J (2004). Managing soil microorganisms to improve productivity of agro-ecosystems. Crit. Rev. Plant Sci..

[8] Xuan DT (2012). Different crop rotation systems as drivers of change in soil bacterial community structure and yield of rice, *Oryza sativa*. Biol. Fertil. Soils.

[9] Huang M (2019). Yield performance of machine-transplanted double-season rice grown following oilseed rape. Sci. Rep..

[10] Yin X (2020). Nitrospira bacteria in paddy soil reduced by biochar application. Agrosyst. Geosci. Environ..

[11] Geisseler D, Scow KM (2014). Long-term effects of mineral fertilizers on soil microorganisms – A review. Soil Biol. Biochem..

[12] Delgado-Baquerizo M (2016). Carbon content and climate variability drive global soil bacterial diversity patterns. Ecol. Monogr..

[13] Norton, J. M. Nitrification in agricultural soils in *Nitrogen in agricultural systems* (eds. Schepers, J. S. & Raun, W. R.) 173–199 (American Society of Agronomy, 2008).

[14] Norton J, Ouyang Y (2019). Controls and adaptive management of nitrification in agricultural soils. Front. Microbiol..

[15] Huang M, Yang L, Qin H, Jiang L (2014). Fertilizer nitrogen uptake by rice increased by biochar application. Biol. Fertil. Soils.

[16] Smit E (2001). Diversity and seasonal fluctuations of the dominant members of the bacterial soil community in a wheat field as determined by cultivation and molecular methods. Appl. Environ. Microbiol..

[17] Xie Z (2017). Chinese milk vetch improves plant growth, development and ^15^N recovery in the rice-based rotation system of south China. Sci. Rep..

[18] Huang, M. *et al*. Increased soil fertility in a long-term rice oilseed rape cropping system and its potential roles in reducing nitrogen inputs and environmental impacts in *Cropping systems: applications, management and impact* (ed. Hodges, J. G.) 103–113 (Nova Science Publishers, 2017).

[19] Edgar RC (2013). UPARSE: highly accurate OTU sequences from microbial amplicon reads. Nat. Methods.

[20] DeSantis TZ (2006). Greengenes, a chimera-checked 16S rRNA gene database and workbench compatible with ARB. Appl. Environ. Microbiol..

[21] Wang Q, Garrity GM, Tiedje JM, Cole JR (2007). Naive Bayesian classifier for rapid assignment of rRNA sequences into the new bacterial taxonomy. Appl. Environ. Microbiol..

[22] Caporaso JG (2010). QIIME allows analysis of high-throughput community sequencing data. Nat. Methods.

